# Comparison of methods to identify aberrant expression patterns in individual patients: augmenting our toolkit for precision medicine

**DOI:** 10.1186/gm509

**Published:** 2013-11-29

**Authors:** Daniel Bottomly, Peter A Ryabinin, Jeffrey W Tyner, Bill H Chang, Marc M Loriaux, Brian J Druker, Shannon K McWeeney, Beth Wilmot

**Affiliations:** 1 Knight Cancer Institute, Oregon Health and Science University, 3181 SW Sam Jackson Park Road, Portland, OR 97239, USA; 2 Oregon Clinical and Translational Research Institute, Oregon Health and Science University, 3181 SW Sam Jackson Park Road, Portland, OR 97239, USA; 3 Department of Cell & Developmental Biology, Oregon Health and Science University, 3181 SW Sam Jackson Park Road, Portland, OR 97239, USA; 4 Department of Pediatrics, Oregon Health and Science University, 3181 SW Sam Jackson Park Road, Portland, OR 97239, USA; 5 Department of Anatomic Pathology, Oregon Health and Science University, 3181 SW Sam Jackson Park Road, Portland, OR 97239, USA; 6 Division of Hematology & Medical Oncology, Oregon Health and Science University, 3181 SW Sam Jackson Park Road, Portland, OR 97239, USA; 7 Howard Hughes Medical Institute, Oregon Health and Science University, 3181 SW Sam Jackson Park Road, Portland, OR 97239, USA; 8 Division of Bioinformatics and Computational Biology, DMICE, Oregon Health and Science University, 3181 SW Sam Jackson Park Road, Portland, OR 97239, USA; 9 Division of Biostatistics, PHPM, Oregon Health and Science University, 3181 SW Sam Jackson Park Road, Portland, OR 97239, USA

## Abstract

**Background:**

Patient-specific aberrant expression patterns in conjunction with functional screening assays can guide elucidation of the cancer genome architecture and identification of therapeutic targets. Since most statistical methods for expression analysis are focused on differences between experimental groups, the performance of approaches for patient-specific expression analyses are currently less well characterized. A comparison of methods for the identification of genes that are dysregulated relative to a single sample in a given set of experimental samples, to our knowledge, has not been performed.

**Methods:**

We systematically evaluated several methods including variations on the nearest neighbor based outlying degree method, as well as the Zscore and a robust variant for their suitability to detect patient-specific events. The methods were assessed using both simulations and expression data from a cohort of pediatric acute B lymphoblastic leukemia patients.

**Results:**

We first assessed power and false discovery rates using simulations and found that even under optimal conditions, high effect sizes (>4 unit differences) were necessary to have acceptable power for any method (>0.9) though high false discovery rates (>0.1) were pervasive across simulation conditions. Next we introduced a technical factor into the simulation and found that performance was reduced for all methods and that using weights with the outlying degree could provide performance gains depending on the number of samples and genes affected by the technical factor. In our use case that highlights the integration of functional assays and aberrant expression in a patient cohort (the identification of gene dysregulation events associated with the targets from a siRNA screen), we demonstrated that both the outlying degree and the Zscore can successfully identify genes dysregulated in one patient sample. However, only the outlying degree can identify genes dysregulated across several patient samples.

**Conclusion:**

Our results show that outlying degree methods may be a useful alternative to the Zscore or Rscore in a personalized medicine context especially in small to medium sized (between 10 and 50 samples) expression datasets with moderate to high sample-to-sample variability. From these results we provide guidelines for detection of aberrant expression in a precision medicine context.

## Background

The use of functional assays such as the interrogation of patient-derived cancer cells against panels of small interfering RNA (siRNA) duplexes or small molecule inhibitors allows patients who are part of the same disease subgroup to be further stratified based on an assessment of the effect of gene down-regulation on cancer cell viability [1,2]. The advent of precision medicine represents a methodological paradigm shift from traditional detection of differences between experimental groups towards identification of individual events or outliers (for example, individual expression patterns and patient-specific siRNA/drug sensitivities). Although some work has been done characterizing patient-specific dysregulation of pathways [3-6], univariate patient-specific analysis of gene expression has not been thoroughly explored.

Arguably the most common type of analysis procedure applied to mRNA expression experiments is the determination of putative differential expression [7-9]. However, even within specific subgroups of patients with cancer, the same genes are not always dysregulated in the same manner in every specimen. Individual expression patterns can reflect underlying mutation, chromosomal rearrangement and copy number events. This shifts the focus to a different type of analysis procedure: identification of a single sample or small subgroups that have divergent expression from the rest of the group (for example, the detection of candidate oncogenic chromosomal aberrations on the basis of outlier gene expression in prostate cancer [10]). Many procedures have been devised to detect the latter situation with earliest efforts, cancer outlier profile analysis (COPA) [10] and the outlier sum (OS) [11], focused on prioritization after a robust standardization procedure. Others have extended this to robust t or F tests [12-16] or similar procedures [17-20]. Additionally, the problem has also been viewed as one of ‘population’ or proportional differences between two groups [21-23]. Recently, the anti-profile method was developed to look for genes with high variability across samples and used to discriminate colon cancer cases from controls [24]. A limitation of these procedures is that they assume both a control as well as an experimental group though several, including OS, COPA and the very recently described mCOPA [25], will work with only one group. Others have focused on the observation that, in the presence of outlying subgroups of patients for a given gene, the distribution would become bi- or multimodal [26-28]. Effective parameter estimation for such mixture models would require substantial sample sizes thereby limiting these approaches to large, well-defined cohorts. Additionally, general methods originally devised in other fields such as the outlying degree (OD) [29,30] or the gene tissue index [31] can be used in a gene-wise univariate context for finding outlying subgroups. However all of these methods, with the exception of the OD method, provide a ranking of genes for a given cohort, not for a specific sample within the cohort. Searching for outliers or ‘hits’ for a given sample is a common procedure for some types of experiments, such as genome-wide siRNA screens. Two procedures used for these experiments are a Z-transformation (Zscore) or robust Zscore (Rscore) along with a cutoff dictating outlier or hit status [32]. Both methods have been applied to microarray analysis as well. For instance, the Zscore approach was first applied to microarray datasets a decade ago [33] and still is used for sample-specific analyses as implemented in the cBio web portal [34]. We also note that the Rscore is the first step of the COPA and OS methods with outlier status determined empirically [10,11].

Complicating the search for outlying subgroups is the fact that microarrays as well as other high throughput assays can be sensitive to many technical factors [35-39]. In addition, expression differences between samples can be caused by many potentially confounding factors regarding clinical samples such as gender, ethnicity and age as well as differences in tissue or cell preparation. A concrete example of such an effect leading to expression differences among two subgroups is the non-coding RNA XIST, which is highly expressed in females but has almost negligible levels in males [40]. Although effective methods exist to correct both known [41] and unknown [42] factors, it may still be important to consider overall sample dissimilarities when the expression values of single genes are compared between samples and/or groups. This will become an even more important issue as we move towards a precision medicine clinical paradigm where it is likely that sample processing would immediately follow acquisition rather than forming balanced batches (in terms of relevant covariates) that can be randomized.

In this paper, we consider the question of how to detect genes that exhibit aberrant expression for a subset of patients focusing on the situation where the subset contains only a single patient sample. We perform simulations testing the effectiveness of multiple approaches including the widely used Zscore and Rscore as well as weighted and unweighted variants of the OD method. We first evaluate these approaches, simulating a wide variety of conditions, and show that the OD methods have advantages over the other two methods in terms of performance. In addition to the simulations, we examine gene ranking results across methods for exon array leukemia expression data in the context of corresponding functional assay results (siRNA hits performed on samples from the same patients). We show that the OD methods provide more flexibility than the Zscore and Rscore and further show that the OD method performs similarly or better than the Zscore for two analytical use cases relating the expression data to the siRNA results.

## Methods

### Simulations

**Figure 1:**
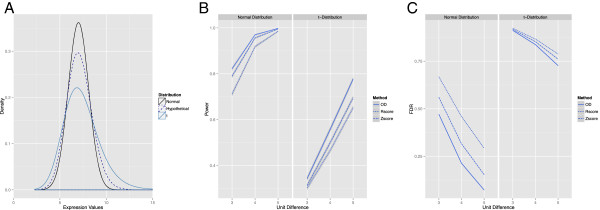
**The outlying degree outperforms other methods in both high and low variability simulated datasets. (A)** Expression data was simulated from two distributions (normal with mean of seven and standard deviation of one as well as a t-distribution with non-centrality parameter set to seven and the degrees of freedom equal to fifteen) that were at the extremes of what would be typically observed in microarray data with the distribution of hypothetical patient data situated somewhere in the middle. **(B, C)** The outlying degree (*k* = 9) significantly outperformed both the Zscore and Rscore method in terms of power and false discovery for all combinations of effect size and distribution type. However, all the methods were only effective when encountering high effect sizes (four to five) with low variability (normal distribution). The grey areas indicate 0.95 confidence intervals. Note that for the false discovery rate, the estimates were very stable and the grey area is not readily observable. OD, outlying degree.

Data were initially simulated from either a normal distribution with mean of seven and standard deviation of one or a t-distribution with 15 degrees of freedom and a non-centrality parameter set to seven. Each data set generated contains 10,000 genes and 20 samples. These distributions and parameters were chosen as they had similar ranges as those from robust multi-array average (RMA) summarized Affymetrix arrays as well as representing the extremes of sample-sample expression variability. These distributions are depicted in Figure 1A along with a hypothetical distribution which would likely mirror that of expression values from a given patient cohort. For a given sample and set of genes, a specified value (three, four or five) was added to introduce the true outlier(s). Similarly, negative two was added to the specified set of genes and samples that were simulated as overall sample technical outliers. The negative value here was chosen because technical outliers more frequently result in decreased intensity values for Affymetrix arrays in our experience. The *P*-value and false discovery rate (FDR) for each statistic was calculated similar to previous work [11,43] as:

(1) $$\text{Pvalu}e_{j}=\frac{\sum_{i\notin G_{1}}^{n}I\left(T_{i}>T_{G_{1}}\right)+\frac{1}{2}\sum_{i\notin G_{1}}^{n}I\left(T_{i}=T_{G_{1}}\right)}{n-1}$$

(2) $$\text{FD}R_{j}=\frac{\sum_{i=1}^{S}I\left(O_{i}\notin G\right)}{S}$$

Where *I*() is the indicator function, *S* is the number of true positive genes (100 in all the simulations), *T* is a vector of length *n* containing the absolute value of the statistics from a given method, and *O* is an ordered version of *T* such that its elements are decreasing. The set *G*_*1*_ is limited to a single integer in (1) whereas *G* is a set of size *S* in (2) representing the position in *T* or *O* respectively with the true outlier gene(s) for a given sample *j*. Note that the FDR in this case assumes 100 true positives, which is meant to simulate an activated pathway. The sample assessed for these statistics was always the one with simulated outlier gene expression. Each different combination of parameters was run 10,000 times. Power was computed as the proportion of the 10,000 iterations which were significant at the .05 level. The FDR was reported as the average FDR observed over the 10,000 iterations for each simulation. Simulations and calculation of the *P*-values and FDR were carried out in parallel on a Beowulf-style cluster using Rmpi [44] with parallel random number generation using L’Ecuyer’s method [45] via the rlecuyer package [46] using R-2.15.1 [47]. Plotting and summaries for the simulations were performed using ggplot2 [48] on R-3.0.1 [47]. Example code for performing these simulations is provided in Additional file 1.

### RNA sample preparation and array processing

All research was performed according to the guidelines of the Helsinki Declaration. Specifically, both oral and written informed consent was obtained from the patient or parent/legal guardian for inclusion in the study. Assent was also obtained for patients between ages 7 and 17 years. The study was reviewed and approved by the Institutional Review Board of Oregon Health & Science University.

Mononuclear cells from blood or bone marrow of patients with hematologic malignancies were isolated using a Ficoll gradient. Cell pellets were snap-frozen in liquid nitrogen and cryopreserved at -80°C for subsequent batch extraction of RNA. RNA was extracted using Qiagen RNeasy kits (Qiagen, Valencia, CA) according to the manufacturer instructions, including performance of the optional on-column DNase treatment step. Samples were amplified and labeled using the Ambion WT Expression/Affymetrix WT Terminal Labeling protocol (Affymetrix, Santa Clara, CA). Amplified and labeled cDNA target samples were each hybridized to a Human Exon 1.0 ST array (Affymetrix, Santa Clara, CA). Image processing was performed using Affymetrix Command Console (AGCC) v.3.1.1 software. The expression data have been deposited in the Gene Expression Omnibus database under the identifier [GEO:GSE42731]. Twelve samples had acceptable RNA integrity number scores (>8) and similar overall intensity distributions and were analyzed further. These samples were processed using the oligo [49] Bioconductor package [50]. Background correction and normalization performed via the RMA method [51] using the core metaprobesets as well as probesets. Note that probeset-level summarizations were used for the visualizations whereas metaprobeset-level summarizations were used for the methods comparisons. Here we consider metaprobesets to denote single transcripts or genes. Ensembl annotations and coordinates were retrieved from Ensembl Build 69 [52] and used in conjunction with the GenomicFeatures package [53] to provide gene contexts for the (meta)probesets. All of the applied analysis was performed using R-3.0.1 [47] with plotting again performed using ggplot2 [48] as well as GenomeGraphs [54]. The reshape2 [55] and biomaRt [56] packages were also utilized.

### Expression prioritization approaches

Let *x*_*ij*_ be the expression or simulated expression data at gene *i* = 1,…,*n* and sample *j* = 1,…,*m*. A commonly used way to screen for outliers is the Zscore, which is the *X* matrix mean centered and scaled by the standard deviation (*sd*):

(3) $$\text{Zscor}e_{\text{ij}}=\frac{x_{\text{ij}}-\text{mean}\left(x_{i}\right)}{\text{sd}\left(x_{i}\right)}$$

where *x*_*i*_ indicates application of the function to the entire vector of sample expression values for gene *i.* Similar to the Zscore method, we define a robust standardization (Rscore) of the *X* matrix as described previously [10]:

(4) $$\text{Rscor}e_{\text{ij}}=\frac{x_{\text{ij}}-\text{median}\left(x_{i}\right)}{\text{mad}\left(x_{i}\right)}$$

where the *mad* function is the median absolute deviation.

The OD method [29,30], is a simple, distance-based method for assigning a score to a given sample indicating the degree with which it differs from the *k* nearest samples for a given gene. For both the OD method and weighted variants of the outlying degree (WOD), we first define an expression distance matrix *D* of dimension *n* × *m-1* containing the absolute values of the pairwise expression differences between the current sample *j* and the remaining samples in the cohort indexed by *j’* for gene *i.* That is, each element of the *D* matrix denoted as *d*_*ij’j*_ is defined as:

(5) dij'j=xij−xij'forj'≠j

For each gene, *i*, the absolute expression differences *d*_*ij’j*_ are sorted in increasing order giving the corresponding *j’* values provided in *o*_*ij*_, which is indexed by *l* taking values from 1 to *m-1*. The outlying degree value for each sample and each gene, *OD*_*ij*_, is the sum of the first *k* elements of the *d*_*ij*_ vector ordered by the *o*_*ij*_ vector as is shown in (6) and (7)*.*

(6) $$o_{\text{ij}}=j{'}_{1}\ldots j{'}_{m-1}\;\text{such}\;\text{that}\;d_{\text{ij}{'}_{1}j}<\ldots <d_{\text{ij}{'}_{m-1}j}$$

(7) ODij=∑l=1kdij'j|j'=oijl

The WOD methods are similar to the OD method but rely on a weight matrix describing the sample-to-sample dissimilarity, which is the *m × m* matrix *W* and is defined by the Euclidean distances between samples as in (8).

(8) wjj'=∑i=1n|xij−xij'|2

The simplest variation of the OD is WODa, which weights each of the *k* nearest distances by the scaled Euclidean distance between the two samples in question. That is, the weights are applied after determining the nearest absolute expression differences.

(9) WODaij=∑l=1kwjj'dij'j|j'=oijl∑l=1kwjj'|j'=oijl

By contrast, the WODb approach first applies the scaled weights, computes the nearest absolute expression differences and then finds the sum of the *k* nearest weighted differences. One difference between (9) and (11) is that the value used to scale the weights is based on the sum of the weights associated with the *k* nearest differences in (9) and the sum of the non-diagonal weights in (11).

(10) owij=j'1…j'm−1suchthatwjj'1dij'1j∑j'≠jwjj'<…<wjj'm−1dij'm−1j∑j'≠jwjj'

(11) WODbij=∑l=1kwjj'dij'j|j'=owijl∑j'≠jwjj'

For all the OD methods, *k* was set to nine or six for the simulated and real data respectively, based on the simulations in Figures S3 and S4 in Additional file 2. An implementation of these methods is provided in Additional file 1 and will be provided as part of an R package ‘pod’ at [57].

## Results and discussion

### Methods and parameters

The Zscore as defined (see Methods) is a simple approach to assessing whether an outlier exists in a moderately sized dataset [58]. However, its use of the difference from the mean as the numerator (as well as the standard deviation in the denominator) means that it potentially could be influenced by outliers itself*.* This is a well-known property of related procedures based on means and many alternatives exist to reduce the influence of outliers, such as the use of trimmed means or medians. The median-based robust analogue of the Zscore utilizes the difference from the median divided by the median absolute deviation (Rscore) as has been suggested in some of the initial work in looking for genomic outliers [10]. The OD, as implemented (see Methods), is a measure of how different the expression value for a given sample is from the expression values from the *k* nearest samples for a given gene. The choice of the *k* parameter in this respect is important as it may impact sensitivity and specificity. The *k* parameter can take integer values between 1 and *m −1* (assuming *m >1*) with the case of *k = 1* equivalent to the absolute difference between the given sample and the most similar of the remaining samples for a given gene*.* For the case of finding genes containing single sample outliers, we carried out several simulations examining both power and FDR for a wide range of *k* values*.* For our simulation size of 20 samples, we found that *k* = 9 seemed to provide good performance over a range of effect sizes (Figures S1 and S2 in Additional file 2) with relatively little additional performance gains above nine. In general a *k* value set to a value near *m*/2 seemed to provide adequate performance for cohort sizes >10 (Figures S3 and S4 in Additional file 2). Note that this assumes that the conditions of the simulation roughly approximate that of the dataset in question and that one is mainly interested in finding single sample outliers. This is likely to be the case for the simulations as they were carried out using similar parameters. Utilizing a different *k* value may influence power and FDR estimates for a given simulation, though from these simulations it appears that decreases in performance would mainly occur when utilizing a substantially lower *k* value. Although performance is difficult to assess using experimental data, we argue that for detection of single sample outliers it is similar enough due to the RMA preprocessing, which makes the overall expression distributions more comparable to each other as well as having a range of expression values similar to the simulations.

### Evaluation using simulations

Several aspects of the OD method could be improved based on an examination of actual array experiments. First, overall dissimilarities between samples could inappropriately increase the score for a given gene, making it desirable to down-weight sample-sample differences based on a measure of overall dissimilarity. An example of this would be an array that had a subset of genes with dissimilar hybridization characteristics but not to the extent that it would be removed for quality control purposes. Also, this would be important in a precision medicine context as we would expect samples to vary in similarity based on technical and biological factors. A straightforward adaptation of the OD method would be to incorporate weights that would decrease the influence on sample-sample comparisons for a given gene if the samples themselves were highly dissimilar. Based on previous work in the field of spatial statistics [59], we implemented several variants of the weighted OD, the only difference being whether the weighting was taken into account before (WODb) or after (WODa) the nearest neighbors were computed.

We first compared all methods using a straightforward power simulation where a single gene had a single sample outlier with a true effect size ranging from three to five units, and where data were either generated from a re-centered normal or t-distribution to capture the range of sample-sample variability to which actual samples might belong (Figure 1A). Weighting the OD method based on overall sample dissimilarity in this context had no benefit over the basic OD approach as all samples would be overall very similar as a product of the simulation approach. However, the OD methods had significantly higher power than either the Zscore or Rscore in all six simulations (Figure 1B). Even for the normal distribution simulation, large effect sizes of four or five were necessary to reach high power (>0.9) for all methods whereas only the OD method achieved adequate power (>0.8) at the lowest evaluated effect size. For the t-distribution, no method was able to achieve adequate power even at the highest effect size. An analogous simulation addressing the FDR was also carried out, which demonstrated that the OD method overall had lower FDR values (all six were significantly lower than Zscore or Rscore; Figure 1C). For both distribution types, the FDR was high especially for an effect size of three. The OD method was the only one to achieve acceptable FDR (<0.10) at an effect size of five for the normal distribution. Together, this indicates that reasonable power should be achievable for experimental samples at the expense of higher false discovery for effect sizes greater than four. As discussed below, effect sizes of this magnitude are observable in expression datasets.

**Figure 2:**
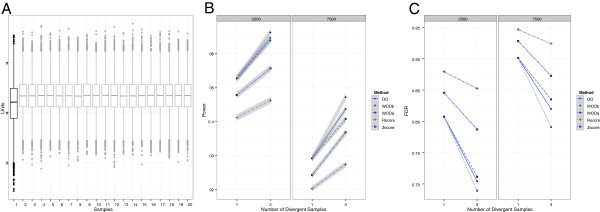
**The weighted outlying degree can attenuate the effect of sample-specific technical variability. (A)** An example of a simulated dataset from the normal distribution with a technical factor affecting 2,500 of the 10,000 genes of sample one, making it divergent. The size of the effect is a two-unit decrease. **(B, C)** display power and false discovery rate estimates for the methods based on similar simulations to **(A)**, where either 2,500 or 7,500 genes of one or three samples were affected. The effect size was kept at five units. The WODb method outperforms the others at least for the case where the number of divergent samples was equal to three. The grey areas indicate 0.95 confidence intervals. Note that for the false discovery rate, the estimates were very stable and the grey area is not readily observable. OD, outlying degree method; WODa, weighted outlying degree with weighting performed after nearest neighbor computations; WODb, weighted outlying degree with weighting performed before nearest neighbor computations.

A more realistic case involves one or more samples containing genes with lower expression on average than the remainder of the cohort. This is often seen due to technical issues that affect the overall hybridization characteristics of a given array. We simulated a rather extreme situation where 2,500 or 7,500 genes in one or three samples were affected by such a technical issue and therefore were two units lower on average than the remainder of the samples (Figure 2A). In each case, we considered the situation where the sample and gene(s) with the true outlier effect were among those impacted by the technical factor. Otherwise data were simulated from the normal distribution with an effect size of five. Overall, the OD methods had significantly higher power and lower FDR values in all four simulations (Figure 2B,C). Differences between the three OD variants were observed when there were three affected samples with the WODb variant having additional performance gains over the other two methods. In all cases, performance was seriously hampered by the introduction of the technical factor, meaning that that these procedures will only perform adequately if all samples are overall similar.

### Evaluations using experimental data

**Figure 3:**
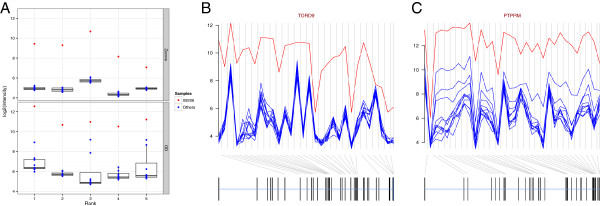
**The outlying degree is more robust to variability across samples than the Zscore in experimental data. (A)** The top five genes for both the Zscore and outlying degree method were found for sample 09206. From comparison purposes we plotted the distribution of the expression levels of the 12 patient samples for the top five ranked genes in either method. It was clear that the Zscore ranked higher those genes where a single outlier was found with the remainder of the samples tightly grouped together whereas the outlying degree (*k =* 6) ranked higher those genes with large differences while tolerating more expression variability between the samples. Exon-level summary of the genes ranked the highest in **(A)** (Rank 1) are shown for both **(B)** the Zscore and **(C)** the outlying degree methods.

We next applied all five methods to an experimental dataset consisting of samples from 12 pediatric patients with acute B lymphoblastic leukemia run on Affymetrix Exon arrays (see Methods). For the OD methods we set *k* to six. We first determined the number of genes that roughly fell into our simulation effect size categories of three, four and five. This was done by computing the difference between the sample with the highest gene expression value and the sample with the second highest gene expression for a given gene (note that this is equivalent to using the OD method with *k* = 1 for the sample with the highest gene expression). We refer to this value as the delta and it assumes that there is a single sample that is up-regulated relative to the rest for a given gene, which is the case in the simulations. We found that there were 3, 14 and 55 genes respectively in each of the effect size categories. As the delta is computed per gene and does not convey sample level information we determined the ranks for the patient sample with the highest expression value. Focusing on the genes with delta values of four or greater, the OD methods performed similarly and all ranks were within the top 10 for the given patient sample. In 9 out of 14 cases (64%), the OD method ranked equal to or higher than the Zscore method, and in 10 out of 14 (71%) when compared to the Rscore method. Because the Rscore had relatively poor performance in the simulations, and the weighted variants of the OD method are most useful in cases of large technical differences for multiple samples, we then focused on the comparison between the OD method and the Zscore. To quantify the differences between the two methods, we examined the top 20 genes for patient sample 09206 from the Zscore and OD method and found that, in general, the Zscore method ranked higher those genes with low sample-sample variability outside of a single outlier whereas the OD method (*k* = 6) tolerated greater variability. We quantified this by computing the standard deviation after removing the highest expression value for the top 20 genes from both methods and observed that the median value of this standard deviation from the OD method was 0.411 (range: 0.144 to 1.784) whereas for the Zscore it was 0.174 (range: 0.051 to 0.384; Figure 3 and Table S1 in Additional file 3). As shown in Figure 3, the top ranked genes for the OD and Zscore methods, *PTPRM* and *TDRD9*, exhibited clear gene-level over-expression. We note that knockdown of *PTPRM* has been previously suggested to decrease cell growth and survival in glioblastoma multiforme [60], suggesting its possible inclusion in a future iteration of the siRNA panel. Less seems to be known about *TDRD9*. It should be noted that the *k* parameter provides a mechanism through which the user can control the type of events that are prioritized for a given sample. For example, increasing *k* allows more sample-sample variability and therefore the rankings will be more divergent from the Zscore, decreasing *k* will do the opposite (Figure S5 in Additional file 2). The user can choose *k* based on his/her hypothesis regarding the sample-sample differences, keeping in mind its effect on power and false discovery as discussed above.

As an initial applied analysis, we examined the results of the OD and Zscore in the context of the patient sample T119, which had an siRNA hit for *ROR1* (Table S2 in Additional file 4). We chose patient sample T119 as it had only a single siRNA hit and therefore we could expect some dysregulated genes that were unique to the sample, demonstrating the arguably most common use case for the Zscore. Overexpression of *ROR1* in acute lymphoblastic leukemia samples with the t(1;19) translocation has been previously characterized [61] and it was hypothesized that the resulting fusion of the genes *E2A* (*TCF3*) and *PBX1* halt the development of the progenitor B cells and continue the expression of *ROR1* along with the preBCR complex. ROR1 and the preBCR complex contribute to proliferation and survival through the PI3K, AKT and MEK/ERK pathways. Examining the expression of *E2A* and *PBX1* in our dataset, we found that *E2A* was highly expressed across all samples while *PBX1* was highly expressed in sample T119 with moderate or low expression in the other samples. As a result, *PBX1* was ranked first and second for the Zscore and OD methods respectively for sample T119. It has also been previously suggested by their joint down-regulation following siRNA treatment of the fusion product [62] that *EB-1 (ANKS1B)* and one isoform of *WNT16* were also up-regulated as a consequence of the *E2A-PBX1* fusion. The *ANKS1B* gene was ranked first by the OD method and ninth by the Zscore method, while the *WNT16* gene was ranked 11^th^ by both methods. *ROR1* itself was ranked 28th and 30th for the Zscore and OD methods respectively. This demonstrates that both the OD and Zscore methods are effective at pulling out potential gene expression signatures related to a specific patient’s disease characteristics when divergent from the rest of the cohort.

Another use case is the identification of commonalities among patient samples that share one or more gene targets. In our dataset, the siRNA hit for samples 09206 and 08419 was shared (*TNK1*) so a natural question is whether there are genes that have shared expression dysregulation between the pairs (Table S2 in Additional file 4). As they differ by gender, we first took the step of removing expression differences due to gender. This was done by first fitting a linear model contrasting the genders for all genes and using the resulting matrix of residuals from the model fit as the expression matrix. We then reran the OD and Zscore methods on the matrix of residuals and compared the ranks of the top 20 of each sample using both methods. For the OD method, the *TMPRSS15* gene was shared between the top 10 genes in the two patient samples ranked second and third for 09206 and 08419 respectively. By comparison, the Zscore method applied to both samples did not share any genes in the top 10, in fact the lowest ranked gene for 08419 that was in the top 10 of 09206 ranked 10,424^th^. This demonstrates that the OD method can, in addition to finding the divergently expressed genes for a single sample, identify and prioritize those genes with shared dysregulation between samples with similar functional or clinical phenotypes.

## Conclusions

We have addressed the motivating problem of how to detect patient-specific expression dysregulation events, as well as providing guidelines and considerations for these types of analyses. Our emphasis was on the situation where one sample was an outlier relative to the rest and on small to moderate cohort sizes, which was representative of our cohort of patient samples also analyzed using an siRNA sensitivity screen. We benchmarked several methods, Zscore and Rscore as well as several variants of the OD method, under a variety of conditions including different effect sizes and the introduction of technical noise. We determined that the OD method performed equally well or better than the others in the majority of our simulations in terms of power and FDR. The weighted variants of the OD methods had greater performance when a large amount of technical noise was introduced into the simulations.

When these methods were applied to a set of 12 expression arrays from acute B lymphoblastic leukemia samples, we showed that the OD method ranked the majority of high effect size genes higher or equivalent to Zscore or Rscore. Focusing on the Zscore and OD comparison, we found that the Zscore ranked higher those genes that had low sample-sample variation outside of a single outlier, whereas the OD method was more tolerant of sample-sample variation depending on the choice of *k.* It was further shown that the results of an OD run with *k* = 1 were more similar to Zscore than OD runs with higher *k* values. When examining the expression data in the context of the siRNA hits, we noted that the pattern of hits derived from the siRNA screen could either be unique to a cohort or be similar among multiple members. This implies that related gene expression outliers should either be unique or shared. The OD was able to robustly prioritize such unique and shared genes whereas the Zscore was only effective at finding the former. We note that there are other similar contexts in which these methods may be successfully applied outside of finding genes related to siRNA screens. For instance, one could find genes related to adverse clinical outcomes that affect only one or two subjects in a given small to medium sized cohort.

Here, we focused on the detection of genes containing sample expression values that were up-regulated relative to the remaining samples. The OD method can also be applied for the detection of down-regulated genes, by determining the sign of the difference from the sample in question and the mean or median of the samples for a given gene.

One of the difficulties of focusing on the detection of outliers for a given set of samples is that it is much more difficult to control for potential confounders, because any number of technical or biological factors can impact a given sample in a high throughput expression experiment. One way to address known confounders would be the application of these methods to the residuals from a least squares fit or robust alternative, as we demonstrated through the correction of gender effects. Protecting against unknown confounders as in the surrogate variable analysis method [42] would seem a natural extension to this idea though further research would be necessary.

For our simulations, we assumed that the overall distributions between the samples were highly similar. This assumption is likely to be valid for Affymetrix arrays when RMA [51] preprocessing and summarization is applied due to the default use of quantile normalization [63]. Because RMA requires the arrays to be preprocessed together, it is desirable to have the expression distributions as comparable as possible to ensure the expression estimates are accurate. As the B-ALL dataset described here was processed in a single batch and each sample analyzed relative to other members of the batch, the RMA procedure was utilized. If multiple batches or even single arrays are analyzed together, a variant of RMA, frozen RMA [64], is an alternative.

This work represents a step towards the analysis of patient samples in a personalized or precision medicine context. We found that the OD method was more efficient at the task of prioritizing gene expression outliers than other alternatives. Also, by being able to take into account overall sample dissimilarities, it is better suited to address the issues inherent in such a clinical paradigm where analysis should not ideally wait for sufficient sample accrual before processing and analysis. The OD method provides the user with the ability to potentially detect gene expression dysregulation events shared between several samples. It can be used in relatively small cohorts and has high power in that scenario to detect outlier samples if there is a high effect size and relatively little sample-sample variability. We note that these requirements appear to be satisfied in the dataset examined here. Because of this, the OD can perform well in many situations and provides a robust analytical approach for the detection of patient-specific events.

## Additional files

The following additional data are available with the online version of this paper.

## Abbreviations

COPA: cancer outlier profile analysis; FDR: false discovery rate; fRMA: frozen robust multi-array analysis; OD: outlying degree; OS: outlier sum; RMA: robust multi-array analysis; Rscore: robust Z score; siRNA: small interfering RNA; WODa: weighted outlying degree with weighting performed after nearest neighbor computations; WODb: weighted outlying degree with weighting performed before nearest neighbor computations.

## Competing interests

The authors declare that they have no competing interests.

## Authors’ contributions

BJD and SKM originally conceived the idea. DB devised the study and carried out the analyses. DB and PAR wrote the code and performed background research. BW and SKM provided guidance for the study. DB, SKM and BW wrote the manuscript. JWT, BHC, MML and BJD collected the leukemia samples, performed exon array and siRNA profiling as well as helped draft the manuscript. All authors read and approved the final manuscript.

## Supplementary Material

Additional file 1 Containing documentation and implementation of the described methods and simulations in R. Click here for file

Additional file 2 Contains the supplemental Figures S1-S5. Click here for file

Additional file 3: Table S1 A comparison of ranks between the Zscore and OD methods. Click here for file

Additional file 4: Table S2 A summary of the siRNA hits for the 12 patient samples. Click here for file
