# Supplementary material for: Comparison of methods to identify aberrant expression patterns in individual patients: augmenting our toolkit for precision medicine
Source: Genome Med. 2013 Nov 29;5(11):103. doi: 10.1186/gm509 (PMC3971350; doi:10.1186/gm509)
Supplement: Additional file 1 — Containing documentation and implementation of the described methods and simulations in R. [file gm509-S1.zip › Additional_File_4/run_simulations.pdf]

Demonstration of simulation code

Daniel Bottomly

September 4, 2013

1 Replication of the simulation figures

Here we reproduce parts of the main simulation figures in the paper 'Identifying Individual Aberrant Expression Patterns: Augmenting our toolkit for Precision Medicine' using smaller-scale calculations as opposed to the parallel calculations presented in the paper itself. Instead of the 10,000 iterations we carried out on our cluster we limit ourselves to 250 iterations as a demonstration. The most noticeable effect of this are the larger confidence limits on those plots. The reader is welcome to see the effect of increasing this parameter this by setting `numruns` to a larger value. Most of the simulation code was initially devised by Peter Ryabinin and much of it was inspired by previous work by other groups who are acknowledged in the source code.

1.1 Initial parameters

These are the parameters and objects used for most of the below simulations.

```
> options(stringsAsFactors=FALSE)
> stopifnot(require(ggplot2))
> stopifnot(require(reshape2))
> stopifnot(require(plyr))
> stopifnot(require(Hmisc))
> source("simulation_cluster_base.R")
> source("simulation_cluster_base_multi.R")
> source("simsvamethod_multicat.R")
> source("methods.R")
> totarrays = 20          # total number of samples/arrays
> totgenes = 10000         # total number of genes
> numcontrol = 19         # number of controls (irrelevant if there is no technical factor)
> numunmodgenes = 250     # number of genes effected by unmodeled factor
> unmodfac = 0            # vector of values for the unmodeled factor
> numruns = 250             # Number of simulation runs
> model.list <- list(
+         weight.od.a=list(func=weight.od.a ,
+                 add.params=list(weight.func=function(x) as.matrix(dist(t(x))), od.k=9)),
```

```
+         weight.od.b=list(func=weight.od.b ,
+                 add.params=list(weight.func=function(x) as.matrix(dist(t(x))), od.k=9)),
+          od=list(func=od.matrix , add.params=list( od.k=9)),
+         z.robust=list(func=robust.z, add.params=NULL),
+         z.score=list(func=zscore.matrix, add.params=NULL))
> #We will actually use this instead of the full model.list
> #        to save time
> sub.model.list <- model.list[c("od", "z.score")]
> get.pval.dta <- function(sim.basic)
+ {
+         sim.basic$folddiff <- factor(as.integer(sim.basic$folddiff), ordered=TRUE)
+     sim.basic$p.val <- as.numeric(sim.basic$p.val)
+     sim.basic$model <- factor(sim.basic$model, ordered=FALSE)
+
+     #need this step to ensure it works for all versions of ggplot2
+
+     sum.dta <- ddply(sim.basic, .(folddiff, model), summarise, num.sig=sum(p.val<.05),
+                          num.samps=length(p.val))
+     sum.dta$Power <-  with(sum.dta, num.sig/num.samps)
+     sum.dta$stdErr <- with(sum.dta, sqrt((Power*(1-Power))/num.samps))
+     sum.dta$ymin <- with(sum.dta, Power - 1.96*stdErr)
+     sum.dta$ymax <- with(sum.dta, Power + 1.96*stdErr)
+
+     return(sum.dta)
+ }
> get.fdr.dta <- function(sim.basic.fdr)
+ {
+     sim.basic.fdr$fdr <- as.numeric(sim.basic.fdr$fdr)
+
+     split.fdr <- split(sim.basic.fdr, list(sim.basic.fdr$folddiff, sim.basic.fdr$model))
+     boot.fdr.list <- lapply(split.fdr, function(x)
+         {
+             return(smean.cl.normal(x$fdr, conf.int=.95, na.rm=TRUE))
+         })
+     boot.fdr.dta <- data.frame(do.call("rbind", boot.fdr.list), stringsAsFactors=FALSE)
+     boot.fdr.dta$folddiff <- factor(as.integer(sapply(strsplit(rownames(boot.fdr.dta),
+                              "\\."), "[[", 1)), ordered=TRUE)
+     boot.fdr.dta$model <- factor(as.character(sapply(strsplit(rownames(boot.fdr.dta),
+                              "\\."), function(x) paste(x[2:length(x)], collapse="."))),
+                          ordered=FALSE)
+
+         return(boot.fdr.dta)
+ }
> #if parallel is installed
>
> options(mc.cores=10)
```

```
> set.seed(123, "L'Ecuyer")
```

1.2 Initial Power simulation for Normal distribution

Here we carry out the basic simulation comparing the Zscore and the OD method in the context of the normal distribution used in the manuscript. Note that the basic estimates plotted in the figure do resemble that of figure 1 though the error estimates are much larger indicating the relative instability of the power calculations and why 10,000 iterations were performed in the actual manuscript.

```
> bi.group.combs <- expand.grid(list(samp.num="unaff.sample.1", folddiff=3:5))
> all.param.list <- lapply(1:nrow(bi.group.combs), function(x)
+         {
+                 sub.combs <- bi.group.combs[x,]
+
+                 samp.assign <- data.frame(samp=as.character(sub.combs$samp.num), gene=1,
+                         folddiff=sub.combs$folddiff, stringsAsFactors=FALSE)
+
+                 return(list(totarrays=totarrays, totgenes=totgenes, numcontrol=numcontrol,
+                                 numunmodgenes=numunmodgenes, unmodfac=unmodfac,
+                                 samp.assign=samp.assign, sim.func="rnorm",
+                                 sim.parms=list(sd=1, mean=7)))
+         })
> names(all.param.list) <- paste("totarrays:", totarrays, "-totgenes:", totgenes,
+                                        "-numcontrol:", numcontrol, "-numunmodgenes:",
+                                        numunmodgenes, "-unmodfac:", unmodfac,
+                                        "-folddiff:", bi.group.combs$folddiff,sep="")
> system.time(results <- run.simulation.cluster.multi(all.param.list=all.param.list,
+                         model.list=sub.model.list, numruns=numruns,
+                         sum.func=function(x) return(as.numeric(x["unaff.sample.1"]))))

r    system   elapsed
16495.388    23.690  2096.846

> sim.res <- summarize.simulations(results)
> p.val.res <- get.pval.dta(sim.res)
>
>

> qplot(y=Power, x=folddiff, group=model, linetype=model, data=p.val.res,
+             geom="line", ylab="Power", xlab="Unit Difference") +
+             geom_smooth(aes(ymin=ymin, ymax=ymax), stat="identity") +
+             guides(linetype=guide_legend(title="Method"))
>
```

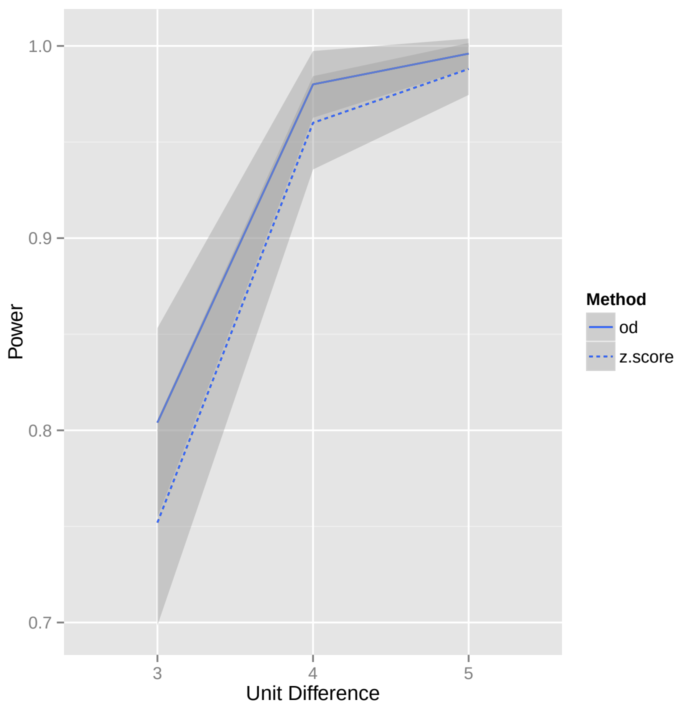

1.3 Initial FDR simulation for Normal distribution

Similar to above we can carry out the simulation assessing the false discovery rate. The only difference here is the creation of 100 true outlier genes as well as the use of `summarize.simulations.fdr` instead of `summarize.simulations` which computes p-values. The same caveats apply as above with regards to the number of iterations though here we see a very similar pattern as that in Figure 1C.

```
> bi.group.combs <- expand.grid(list(samp.num="unaff.sample.1", folddiff=3:5))
> all.param.list <- lapply(1:nrow(bi.group.combs), function(x)
+ {
+         sub.combs <- bi.group.combs[x,]
+
+         samp.assign <- data.frame(samp=as.character(sub.combs$samp.num), gene=1:100,
+                                   folddiff=sub.combs$folddiff, stringsAsFactors=FALSE)
+
+         return(list(totarrays=totarrays, totgenes=totgenes, numcontrol=numcontrol,
+                             numunmodgenes=numunmodgenes, unmodfac=unmodfac,
+                             samp.assign=samp.assign, sim.func="rnorm",
+                             sim.parms=list(sd=1, mean=7)))
+ })
```

```
> names(all.param.list) <- paste("totarrays:", totarrays, "-totgenes:", totgenes,
+                                             "-numcontrol:", numcontrol, "-numunmodgenes:",
+                                             numunmodgenes, "-unmodfac:", unmodfac,
+                                             "-folddiff:", bi.group.combs$folddiff,sep="")
> results <- run.simulation.cluster.multi(all.param.list=all.param.list,
+                         model.list=sub.model.list, numruns=numruns,
+                         sum.func=function(x) return(as.numeric(x["unaff.sample.1"])))
> sim.res.fdr <- summarize.simulations.fdr(results)
> all.fdr.dta <- get.fdr.dta(sim.res.fdr)
>

> qplot(y=Mean, x=folddiff, group=model, linetype=model, data=all.fdr.dta,
+             geom="line", ylab="FDR", xlab="Unit Difference") +
+             geom_smooth(aes(ymin=Lower, ymax=Upper), stat="identity") +
+             guides(linetype=guide_legend(title="Method"))
>
```

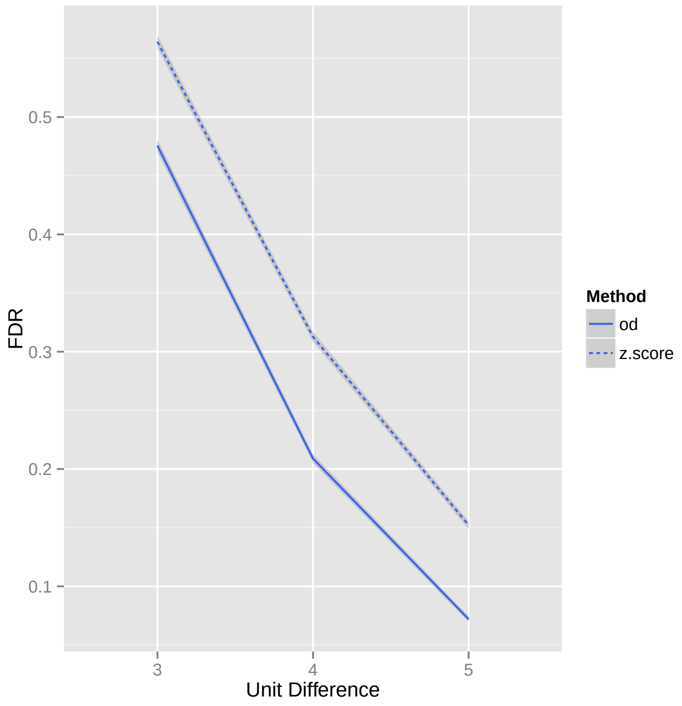

1.4 Simulations for T distribution

To create estimates of FDR and/or power for the T distribution described in the paper, simply set `sim.func` to ”rt” and `sim.parms` to list(df=15, ncp=7).

```
> bi.group.combs <- expand.grid(list(samp.num="unaff.sample.1", folddiff=3:5))
> all.param.list <- lapply(1:nrow(bi.group.combs), function(x)
+         {
+                 sub.combs <- bi.group.combs[x,]
+
+                 samp.assign <- data.frame(samp=as.character(sub.combs$samp.num), gene=1,
+                                          folddiff=sub.combs$folddiff,
+                                          stringsAsFactors=FALSE)
+
+                 return(list(totarrays=totarrays, totgenes=totgenes, numcontrol=numcontrol,
+                                 numunmodgenes=numunmodgenes, unmodfac=unmodfac,
+                                 samp.assign=samp.assign, sim.func="rt",
+                                  sim.parms=list(df=15, ncp=7)))
+         })
> names(all.param.list) <- paste("totarrays:", totarrays, "-totgenes:", totgenes,
+                                             "-numcontrol:", numcontrol, "-numunmodgenes:",
+                                             numunmodgenes, "-unmodfac:", unmodfac,
+                                             "-folddiff:", bi.group.combs$folddiff,sep="")
> results <- run.simulation.cluster.multi(all.param.list=all.param.list,
+                         model.list=sub.model.list, numruns=numruns,
+                         sum.func=function(x) return(as.numeric(x["unaff.sample.1"])))
> sim.res <- summarize.simulations(results)
> p.val.res <- get.pval.dta(sim.res)
>
>

> qplot(y=Power, x=folddiff, group=model, linetype=model, data=p.val.res,
+             geom="line", ylab="Power", xlab="Unit Difference") +
+             geom_smooth(aes(ymin=ymin, ymax=ymax), stat="identity") +
+             guides(linetype=guide_legend(title="Method"))
>
```

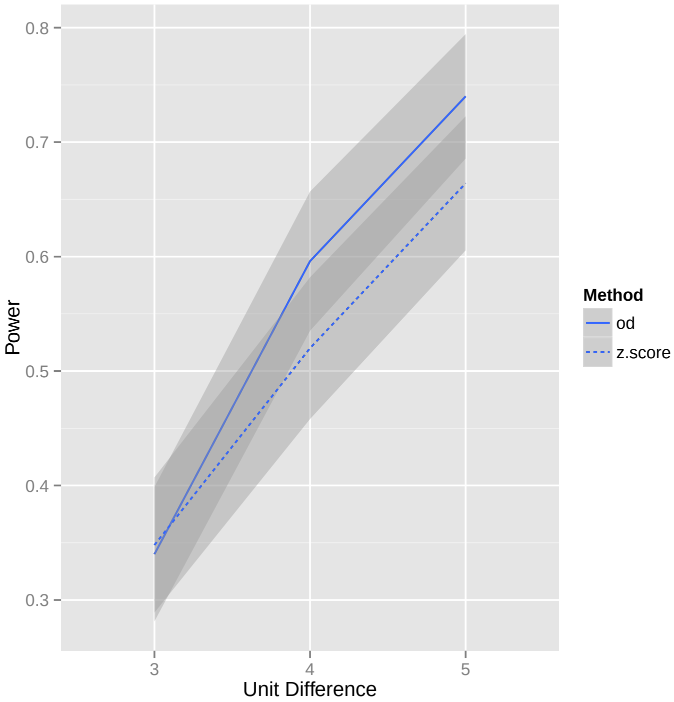

1.5 Assessment of OD weighting to address technical factors

To carry out the assessment of weighting in the context of technical sample outliers we have to ensure that the placement of the true outlier (in terms of sample and gene) overlaps or doesn't overlap with the effect of the techical outlier. In the paper we focused on the situation where there was complete overlap between the technical effect and the true outlier. Below we replicate Fig 2 (b and c) in part using all methods except the Rscore.

```
> sub.model.list <- model.list[c("od", "z.score", "weight.od.a", "weight.od.b")]
> unmodfac <- -2
> bi.group.combs <- expand.grid(list(samp.num="aff.sample.1", folddiff=5, gene="aff",
+          totarrays=20, numunmodgenes=c(2500,7500), numcontrol=c(17,19)))
>  all.param.list <- lapply(1:nrow(bi.group.combs), function(x)
+         {
+             sub.combs <- bi.group.combs[x,]
+
+             samp.assign <- data.frame(samp=as.character(sub.combs$samp.num),
+                     gene=as.character(sub.combs$gene), folddiff=sub.combs$folddiff,
+                 stringsAsFactors=FALSE)
+
```

```
+             return(list(totarrays=sub.combs$totarrays, totgenes=totgenes,
+                              numcontrol=sub.combs$numcontrol,
+                              numunmodgenes=sub.combs$numunmodgenes,
+                          unmodfac=unmodfac, samp.assign=samp.assign,
+                          sim.func="rnorm", sim.parms=list(sd=1, mean=7)))
+         })
>   names(all.param.list) <- paste("totarrays:", bi.group.combs$totarrays,
+                 "-totgenes:", totgenes, "-numcontrol:", bi.group.combs$numcontrol,
+                 "-numunmodgenes:", bi.group.combs$numunmodgenes,sep="")
>    results <- run.simulation.cluster.multi(all.param.list=all.param.list,
+                 model.list=sub.model.list, numruns=numruns,
+                 sum.func=function(x) return(as.numeric(x["aff.sample.1"])))
>    sim.res <- summarize.simulations(results)
>    sum.dta <- ddply(sim.res, .(model, numunmodgenes, numcontrol), summarise,
+                 num.sig=sum(p.val<.05), num.samps=length(p.val))
>    sum.dta$Power <-  with(sum.dta, num.sig/num.samps)
>    sum.dta$stdErr <- with(sum.dta, sqrt((Power*(1-Power))/num.samps))
>    sum.dta$ymin <- with(sum.dta, Power - 1.96*stdErr)
>     sum.dta$ymax <- with(sum.dta, Power + 1.96*stdErr)
>     sum.dta$numcontrol <- factor(sum.dta$numcontrol, levels=c("19", "17"),
+                     labels=c("1", "3"))
>

> qplot(y=Power, x=numcontrol, group=model, color=model, data=sum.dta,
+            geom="line", ylab="Power", xlab="Number of Divergent Samples",
+            facets=~numunmodgenes) +
+           geom_smooth(aes(ymin=ymin, ymax=ymax), stat="identity")
```

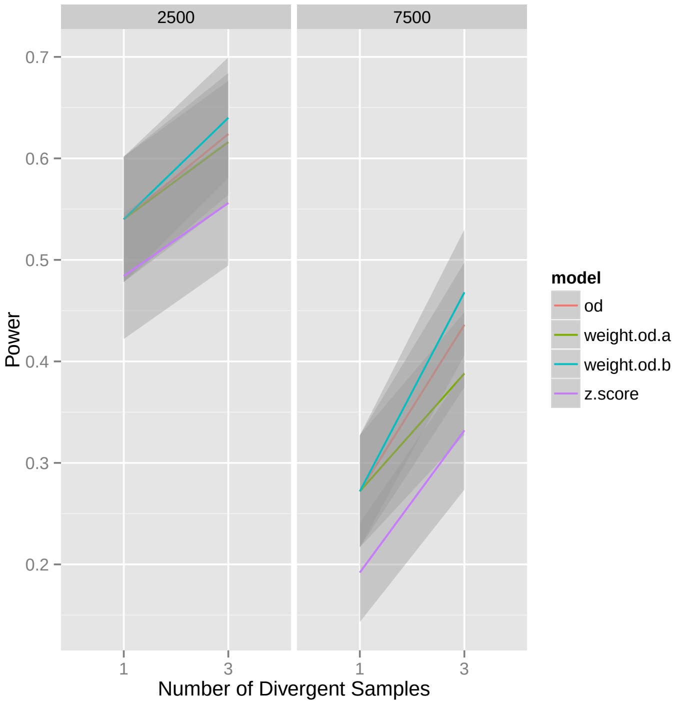

Similarly we will carry out an example of the FDR assessment of the technical factor simulation.

```
> bi.group.combs <- expand.grid(list(samp.num="aff.sample.1", folddiff=5, gene="aff",
+                 totarrays=20, numunmodgenes=c(2500,7500), numcontrol=c(17,19)))
>  all.param.list <- lapply(1:nrow(bi.group.combs), function(x)
+          {
+              sub.combs <- bi.group.combs[x,]
+
+              samp.assign <- data.frame(samp=as.character(sub.combs$samp.num),
+                      gene=rep(as.character(sub.combs$gene), 100),
+                  folddiff=sub.combs$folddiff,
+                  stringsAsFactors=FALSE)
+
+              return(list(totarrays=sub.combs$totarrays, totgenes=totgenes,
+                      numcontrol=sub.combs$numcontrol,
+                      numunmodgenes=sub.combs$numunmodgenes, unmodfac=unmodfac,
+                      samp.assign=samp.assign, sim.func="rnorm",
+                      sim.parms=list(sd=1, mean=7)))
+          })
>          names(all.param.list) <- paste("totarrays:", bi.group.combs$totarrays,
+                          "-totgenes:", totgenes, "-numcontrol:", bi.group.combs$numcontrol,
```

```
+                  "-numunmodgenes:", bi.group.combs$numunmodgenes,sep="")
>         results <- run.simulation.cluster.multi(all.param.list=all.param.list,
+                 model.list=sub.model.list, numruns=numruns,
+                 sum.func=function(x) return(as.numeric(x["aff.sample.1"])))
>         sim.res <- summarize.simulations.fdr(results)
>         split.fdr <- split(sim.res, list(sim.res$numunmodgenes, sim.res$numcontrol,
+                 sim.res$model))
>     boot.fdr.dta <- data.frame(t(sapply(split.fdr, function(x)
+         {
+             return(smean.cl.normal(as.numeric(x$fdr), conf.int=.95, na.rm=TRUE))
+         })), stringsAsFactors=FALSE)
>     boot.fdr.dta$numunmodgenes <- factor(as.integer(sapply(strsplit(rownames(boot.fdr.dta),
+                     "\\."), "[[", 1)), ordered=TRUE)
>     boot.fdr.dta$numcontrol <- factor(as.integer(sapply(strsplit(rownames(boot.fdr.dta),
+                     "\\."), "[[", 2)), ordered=TRUE)
>     boot.fdr.dta$model <- factor(as.character(sapply(strsplit(rownames(boot.fdr.dta),
+                     "\\."), function(x) paste(x[3:length(x)], collapse="."))),
+                 ordered=FALSE)
>     rownames(boot.fdr.dta) <- NULL
>     boot.fdr.dta$numcontrol <- factor(as.character(boot.fdr.dta$numcontrol),
+                     levels=c("19", "17"),  labels=c("1", "3"))
>

> qplot(y=Mean, x=numcontrol, group=model, color=model, data=boot.fdr.dta,
+            geom="line", ylab="FDR", xlab="Number of Divergent Samples",
+            facets=~numunmodgenes) +
+          geom_smooth(aes(ymin=Lower, ymax=Upper), stat="identity")
>
>
```

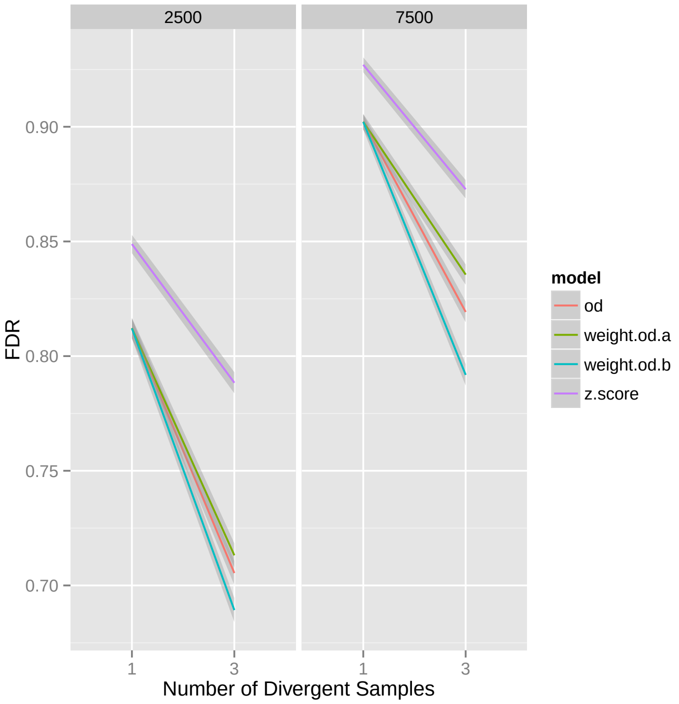

1.6 Applied analysis setup

Now we will carry out the applied analysis assuming the CEL files are downloaded and within a local folder which we will call 'cel.dir'. This example will not run unless 'cel.dir' is modified to be an appropriate directory containing the specified CEL files. Upon publication, these CEL files shall be in GEO as indicated in the manuscript.

```
> library(GenomeGraphs)
> library(GenomicFeatures)
> library(oligo)
> library(pd.huex.1.0.st.v2)
> library(org.Hs.eg.db)
> cel.dir <- "../../expression/432BD_CELS"
> stopifnot(file.exists(cel.dir))
> #make probeset-level summaries
>
> sampleNames <- c('09206', '09788', '09562', '09177', '09182', '09624', '08431', 'T119',
+                                  '09076', '08419', '08118', '09190')
> filenames <- c('03A_A11H1_09206_432BD.CEL', '02A_A11H1_09788_432BD.CEL',
+          '013A_A11H1_09562_432BD.CEL', '05A_A11H1_09177_432BD.CEL',
+             '08A_A11H1_09182_432BD.CEL', '012A_A11H1_09624_432BD.CEL',
```

```
+          '011A_A11H1_08431_432BD.CEL', '010A_A11H1_T119_432BD.CEL',
+             '014A_A11H1_09076_432BD.CEL', '06A_A11H1_08419_432BD.CEL',
+         '07A_A11H1_08118_432BD.CEL', '015A_A11H1_09190_432BD.CEL')
> affy.exprs <- read.celfiles(filenames=file.path(cel.dir, filenames),
+         sampleNames=sampleNames, pkgname="pd.huex.1.0.st.v2")

Reading in : ../../expression/432BD_CELS/03A_A11H1_09206_432BD.CEL
Reading in : ../../expression/432BD_CELS/02A_A11H1_09788_432BD.CEL
Reading in : ../../expression/432BD_CELS/013A_A11H1_09562_432BD.CEL
Reading in : ../../expression/432BD_CELS/05A_A11H1_09177_432BD.CEL
Reading in : ../../expression/432BD_CELS/08A_A11H1_09182_432BD.CEL
Reading in : ../../expression/432BD_CELS/012A_A11H1_09624_432BD.CEL
Reading in : ../../expression/432BD_CELS/011A_A11H1_08431_432BD.CEL
Reading in : ../../expression/432BD_CELS/010A_A11H1_T119_432BD.CEL
Reading in : ../../expression/432BD_CELS/014A_A11H1_09076_432BD.CEL
Reading in : ../../expression/432BD_CELS/06A_A11H1_08419_432BD.CEL
Reading in : ../../expression/432BD_CELS/07A_A11H1_08118_432BD.CEL
Reading in : ../../expression/432BD_CELS/015A_A11H1_09190_432BD.CEL

> norm.exprs.fs.probeset <-  rma(affy.exprs, target="probeset")

Background correcting
Normalizing
Calculating Expression

> norm.exprs.probeset <-  exprs(norm.exprs.fs.probeset)
> #meta-probeset level summaries
>
> norm.exprs.fs.meta <- rma(affy.exprs, target="core")

Background correcting
Normalizing
Calculating Expression

> norm.exprs.meta <-  exprs(norm.exprs.fs.meta)
>
```

Utilizing the GenomicFeatures and annotation packages, below is how the metaprobesets were assigned to genes assuming the coordinates in the platform design database are accurate. Note the database ensembl_human_db.sqlite was created using the GenomicFeatures package as follows:

```
> hsaps.ens <- makeTranscriptDbFromBiomart(biomart="ensembl",
+         dataset="hsapiens_gene_ensembl")
> saveDb(hsaps.ens, file="ensembl_human_db.sqlite")
>
```

```
> hsaps.ens <- loadDb("../../expression/ensembl_human_db.sqlite")
> .mps.to.symbol <- function(mps.ids, hsaps.ens)
+ {
+     mps.list <- lapply(mps.ids, function(x)
+         {
+             print(x)
+             probe.matches <- dbGetQuery(pd.huex.1.0.st.v2@getdb(),
+             paste("select * from core_mps join featureSet using (fsetid) join",
+             "chrom_dict using (chrom) where meta_fsetid = ",x))
+
+             if (nrow(probe.matches) == 0)
+             {
+                 return(NULL)
+             }
+
+             temp.ranges <- GRanges(seqnames=Rle(sub("chr", "", probe.matches$chrom_id)),
+                     ranges=IRanges(start=probe.matches$start, end=probe.matches$stop),
+                  strand="*")
+
+             temp.trans <- transcriptsByOverlaps(hsaps.ens, temp.ranges,
+                     columns=c("tx_name", "gene_id"))
+
+             unique.ens <- unique(unlist(values(temp.trans)$gene_id))
+
+             symb.table <- toTable(org.Hs.egSYMBOL)
+             ens.table <- toTable(org.Hs.egENSEMBL2EG)
+
+             ens.to.symb <- merge(ens.table, symb.table, by="gene_id", all.x=TRUE,
+                     incomparables=NA, sort=FALSE)
+             gene.name <- ens.to.symb[ens.to.symb$ensembl_id %in% unique.ens,]
+             if (nrow(gene.name) == 0)
+             {
+                 return(NULL)
+             }
+             return(cbind(mps=x, gene.name))
+         })
+
+     return(data.frame(do.call("rbind", mps.list), stringsAsFactors=FALSE))
+ }
>
```

1.7 Summary stats for high delta genes

```
> max.deltas <- apply(norm.exprs.meta, 1, function(x)
+           {
+                 ord.x <- sort(x, decreasing=TRUE)
```

```
+                  diff.x <- ord.x[1] - ord.x[2]
+                  return(diff.x)
+            })
> sum(max.deltas > 5)

[1] 3

> sum(max.deltas > 4)

[1] 14

> sum(max.deltas > 3)

[1] 55

> model.list <- list(weight.od.a=list(func=weight.od.a ,
+                                add.params=list(weight.func=function(x) as.matrix(dist(t(x
+                                od.k=6)),
+                        weight.od.b=list(func=weight.od.b ,
+                                add.params=list(weight.func=function(x) as.matrix(dist(t(x
+                                od.k=6)),
+                        od=list(func=od.matrix ,
+                                        add.params=list( od.k=6)),
+                        z.robust=list(func=robust.z, add.params=NULL),
+                        z.score=list(func=zscore.matrix, add.params=NULL))
> model.res.ord.list <- lapply(model.list, function(y)
+         {
+                 rank.mat <- y$func(norm.exprs.meta, y$add.params)
+                 sapply(colnames(rank.mat), function(x) order(rank.mat[,x],
+                  decreasing=TRUE))
+         })
> exprs.max.deltas <- norm.exprs.meta[order(max.deltas, decreasing=TRUE),]
> top.ranks <- t(sapply(1:sum(max.deltas>=4), function(x)
+            {
+                 top.samp <- names(sort(exprs.max.deltas[x,], decreasing=TRUE))[1]
+ delta.val <- which(rownames(norm.exprs.meta) == rownames(exprs.max.deltas)[x])
+                 sapply(model.res.ord.list, function(y) which(y[,top.samp] == delta.val))
+            }))
> sum(top.ranks[,"od"] <= top.ranks[,"z.score"])

[1] 9

> sum(top.ranks[,"od"] <= top.ranks[,"z.robust"])

[1] 10

>
```

1.8 OD and Zscore ranking for sample 09206

```
> top.od <- model.res.ord.list[["od"]][1:20,"09206"]
> pos.in.zscore <- match(top.od, model.res.ord.list[["z.score"]][,"09206"])
> od.deltas <- apply(norm.exprs.meta[top.od,], 1, function(x)
+          {
+               ord.x <- sort(x, decreasing=TRUE)
+               diff.x <- ord.x[1] - ord.x[2]
+               return(diff.x)
+          })
> od.jsd <- apply(norm.exprs.meta[top.od,], 1, function(x)
+               {
+                    sort.x <- sort(x, decreasing=TRUE)
+                    return(sd(sort.x[-1]))
+               })
> od.tab <- data.frame(mps=rownames(norm.exprs.meta)[top.od], rank=1:length(top.od),
+         rank.in.zscore=pos.in.zscore, delta=as.numeric(od.deltas), jsd=od.jsd,
+         stringsAsFactors=FALSE)
> od.symbs <- .mps.to.symbol(rownames(norm.exprs.meta)[top.od], hsaps.ens)

[1] "3777470"
[1] "2345023"
[1] "3926271"
[1] "3404530"
[1] "3367673"
[1] "3553998"
[1] "3067478"
[1] "3491486"
[1] "2680046"
[1] "2736060"
[1] "2779527"
[1] "3404549"
[1] "3777770"
[1] "2519294"
[1] "2533670"
[1] "3349293"
[1] "2822215"
[1] "3617574"
[1] "2701109"
[1] "2729667"

> od.annots <- data.frame(do.call("rbind", lapply(split(od.symbs,od.symbs$mps),
+         function(x) return(cbind(mps=unique(as.character(x$mps)),
+         symbol=paste(unique(x$symbol), collapse=","))))), stringsAsFactors=FALSE)
> od.merged <- merge(od.tab, od.annots, by="mps", sort=FALSE)
> od.merged
```

```
      mps rank rank.in.zscore      delta       jsd              symbol
1  3777470    1             50 3.63034264 0.8403326               PTPRM
2  2345023    2              3 4.59884624 0.1821723               CLCA1
3  3926271    3            180 1.79040246 1.5146695            TMPRSS15
4  3404530    4             16 4.09375226 0.3839816             CLEC12A
5  3367673    5            176 2.04358608 1.3903628              MPPED2
6  3553998    6              1 4.20258030 0.1438965               TDRD9
7  3067478    7              2 4.25274534 0.1650514               NRCAM
8  3491486    8             14 3.83073095 0.3225701              PCDH17
9  2680046    9             10 3.68013984 0.2281736 ADAMTS9,ADAMTS9-AS2
10 2736060   10              7 3.60298645 0.1952038               GRID2
11 2779527   11             18 3.39178491 0.3401995              DDIT4L
12 3404549   12              8 3.48442376 0.2084178      CLEC1B,CLEC12B
13 3777770   13              4 3.51071368 0.1541888               PTPRM
14 2519294   14             47 2.78778334 0.5412217             FAM171B
15 2533670   15             36 3.18437669 0.4370800               AGAP1
16 3349293   16            155 0.90956079 0.9735837  NCAM1,LOC100288346
17 2822215   17             49 2.17879381 0.5085638                 PAM
18 3617574   18          21868 0.07807266 1.7838344     GOLGA8A,GOLGA8B
19 2701109   19            208 1.81095806 1.1699477      MED12L,IGSF10
20 2729667   20             80 2.41280963 0.6652238               STAP1
```

```
> top.zscore <- model.res.ord.list[["z.score"]][1:20,"09206"]
> pos.in.od <- match(top.zscore, model.res.ord.list[["od"]][,"09206"])
> zscore.deltas <- apply(norm.exprs.meta[top.zscore,], 1, function(x)
+           {
+                 ord.x <- sort(x, decreasing=TRUE)
+                 diff.x <- ord.x[1] - ord.x[2]
+                 return(diff.x)
+           })
> zscore.sd <- apply(norm.exprs.meta[top.zscore,], 1, function(x)
+               {
+                     sort.x <- sort(x, decreasing=TRUE)
+                     return(sd(sort.x[-1]))
+               })
> zscore.tab <- data.frame(mps=rownames(norm.exprs.meta)[top.zscore],
+         rank=1:length(top.zscore), rank.in.od=pos.in.od, delta=as.numeric(zscore.deltas),
+         jsd=zscore.sd, stringsAsFactors=FALSE)
> zscore.symbs <- .mps.to.symbol(rownames(norm.exprs.meta)[top.zscore], hsaps.ens)
```

```
[1] "3553998"
[1] "3067478"
[1] "2345023"
[1] "3777770"
[1] "3292169"
[1] "2870964"
```

```
[1] "2736060"
[1] "3404549"
[1] "2739468"
[1] "2680046"
[1] "3449068"
[1] "2590736"
[1] "2738664"
[1] "3491486"
[1] "3359121"
[1] "3404530"
[1] "2727226"
[1] "2779527"
[1] "3489138"
[1] "2749484"
```

```
> zscore.annots <- data.frame(do.call("rbind", lapply(split(zscore.symbs,zscore.symbs$mps),
+          function(x) return(cbind(mps=unique(as.character(x$mps)),
+          symbol=paste(unique(x$symbol), collapse=","))))), stringsAsFactors=FALSE)
> zscore.merged <- merge(zscore.tab, zscore.annots, by="mps", sort=FALSE)
> zscore.merged
```

```
       mps rank rank.in.od     delta        jsd              symbol
1  3553998    1          6 4.2025803 0.14389650               TDRD9
2  3067478    2          7 4.2527453 0.16505141               NRCAM
3  2345023    3          2 4.5988462 0.18217228               CLCA1
4  3777770    4         13 3.5107137 0.15418881               PTPRM
5  3292169    5         73 2.0122114 0.08927948              CTNNA3
6  2870964    6         27 2.8022530 0.13061793            EPB41L4A
7  2736060    7         10 3.6029864 0.19520380               GRID2
8  3404549    8         12 3.4844238 0.20841777      CLEC1B,CLEC12B
9  2739468    9        266 1.2155104 0.07371705               ENPEP
10 2680046   10          9 3.6801398 0.22817358 ADAMTS9,ADAMTS9-AS2
11 3449068   11         23 2.8271200 0.19354145               TMTC1
12 2590736   12         91 1.7750831 0.13419691              NCKAP1
13 2738664   13        101 1.7447348 0.13173758               SGMS2
14 3491486   14          8 3.8307310 0.32257012              PCDH17
15 3359121   15         47 2.2037350 0.18614549                IGF2
16 3404530   16          4 4.0937523 0.38398159             CLEC12A
17 2727226   17        685 0.8336731 0.07883132  PDGFRA,FIP1L1,LNX1
18 2779527   18         11 3.3917849 0.34019949              DDIT4L
19 3489138   19         48 2.2222997 0.21389923             CYSLTR2
20 2749484   20       1962 0.5273304 0.05062609               RXFP1
```

```
> median(od.merged$jsd)
```

```
[1] 0.4105308
```

```
> range(od.merged$jsd)
```

```
[1] 0.1438965 1.7838344

> median(zscore.merged$jsd)

[1] 0.1736118

> range(zscore.merged$jsd)

[1] 0.05062609 0.38398159

>
```

1.9 Generation of Figure S5

```
> library(GeneSelector)
> od.list <- lapply(seq(1, 12, by=3), function(x) od.matrix(norm.exprs.meta,
+         add.params=list(od.k=x)))
> names(od.list) <- paste("OD", seq(1, 12, by=3), sep=".")
> #add zscore as a reference
>
> od.list <- append(list(Zscore=zscore.matrix(norm.exprs.meta)), od.list)
> #note that GeneSelector uses rank(-abs(statistic)) as in:
> #showMethods(RankingFoxDimmic, includeDefs=TRUE)
>
> method.ord.list <- lapply(names(od.list), function(y)
+ {
+         return(new("GeneRanking", ranking=rank(-abs(od.list[[y]][,"09206"])), method=y))
+ })
> merged.list <- MergeMethods(method.ord.list)
>

> HeatmapRankings(merged.list, ind=1:100)
>
```

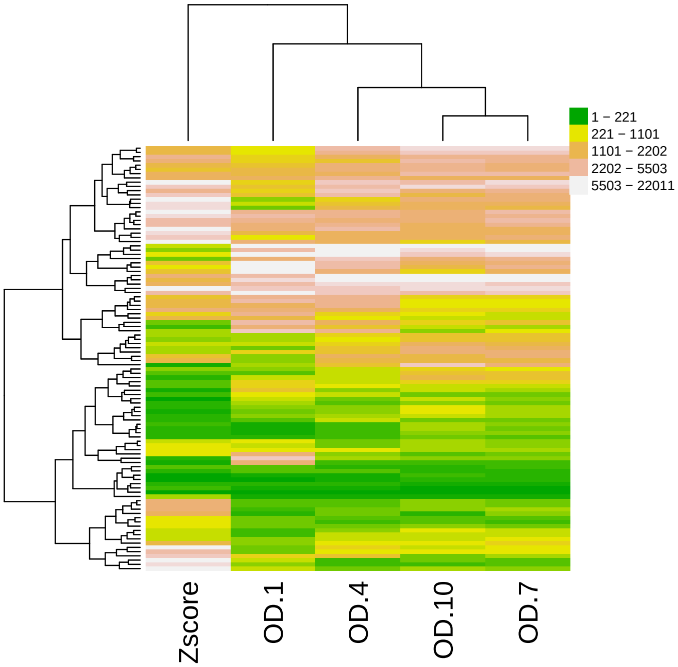

1.10 Generation of Figure 3

```
> qplot(x=rank, y=value,data=all.method, group=rank, geom="boxplot",
+         outlier.shape=NA, facets=type~., xlab="Rank",
+         ylab="log2(Intensity)") + geom_point(aes(color=highlight)) +
+         scale_color_manual(values=c("red", "blue"),name="Samples")
>
```

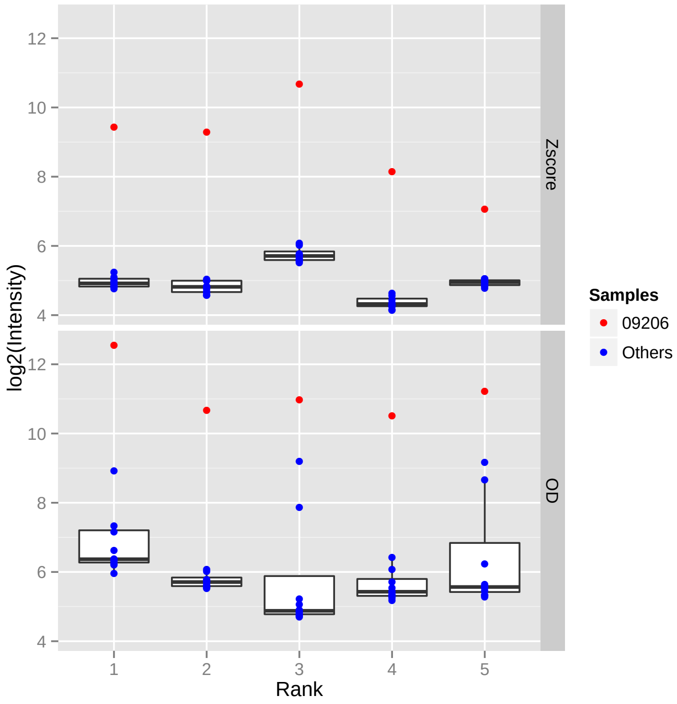


```
> top.mps <- rownames(norm.exprs.meta)[c(zscore.ord[1], od.ord[1])]
> names(top.mps) <- c("zscore", "od")
> top.pat <- "09206"
> ex.list <- exonsBy(hsaps.ens, "tx", use.name=TRUE)
> trans.list <- transcriptsBy(hsaps.ens, "gene")
> plot.list <- lapply(1:length(top.mps), function(i)
+     {
+                 probe.matches <- dbGetQuery(pd.huex.1.0.st.v2@getdb(),
+
+                 paste("select * from core_mps join featureSet using (fsetid) join",
+                  "chrom_dict using (chrom) where meta_fsetid = ",top.mps[i]))
+
+                 temp.ranges <- GRanges(seqnames=Rle(sub("chr", "",
+                 probe.matches$chrom_id)), ranges=IRanges(start=probe.matches$start,
+                 end=probe.matches$stop), strand="*")
+
+                 temp.trans <- transcriptsByOverlaps(hsaps.ens, temp.ranges,
+                         columns=c("tx_name", "gene_id"))
+
+                 unique.ens <- unique(unlist(values(temp.trans)$gene_id))
+
+                 symb.table <- toTable(org.Hs.egSYMBOL)
```

```
+                  ens.table <- toTable(org.Hs.egENSEMBL2EG)
+
+                  ens.to.symb <- merge(ens.table, symb.table, by="gene_id", all.x=TRUE,
+                          incomparables=NA, sort=FALSE)
+
+                  gene.name <- ens.to.symb[ens.to.symb$ensembl_id %in% unique.ens,"symbol"]
+
+                  title <- makeTitle(text = paste(gene.name, collapse=","),
+                          color = "darkred", size=.5)
+
+                  sub.exs <- ex.list[values(temp.trans)$tx_name]
+
+                  red.exs <- reduce(unlist(sub.exs))
+
+                  gene.mod <- makeGeneModel(start=start(red.exs), end=end(red.exs),
+                          chromosome=as.character(unique(seqnames(red.exs))),
+                          dp=DisplayPars(color="cornflowerblue"))
+
+                  probeset.colors <- rep("blue", ncol(norm.exprs.probeset))
+                  names(probeset.colors) <- colnames(norm.exprs.probeset)
+                  probeset.colors[top.pat] <- "red"
+
+                  exon <- with(probe.matches,
+                          makeExonArray(intensity = norm.exprs.probeset[as.character(fsetid)
+                          probeStart = start, probeEnd=stop, probeId=as.character(fsetid),
+                          nProbes=rep(1, length(start)), dp=DisplayPars(color=probeset.colo
+                          mapColor="lightgrey", probeSetColor = "lightgrey",
+                          probeSetLwd=.5, displayProbesets=FALSE)))
+
+                  return(list(title=title, exon=exon, gene.mod=gene.mod, red.exs=red.exs))
+     })
>
>

> gdPlot(list(plot.list[[1]]$title, plot.list[[1]]$exon, plot.list[[1]]$gene.mod),
+         minBase=min(start(plot.list[[1]]$red.exs)),
+         maxBase=max(end(plot.list[[1]]$red.exs)))
>
```

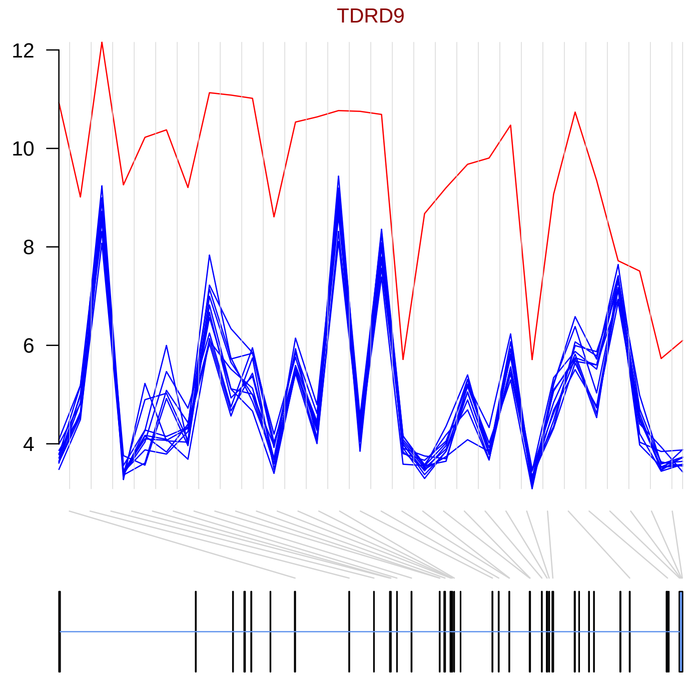


```
> gdPlot(list(plot.list[[2]]$title, plot.list[[2]]$exon, plot.list[[2]]$gene.mod),
+          minBase=min(start(plot.list[[2]]$red.exs)),
+          maxBase=max(end(plot.list[[2]]$red.exs)))
```

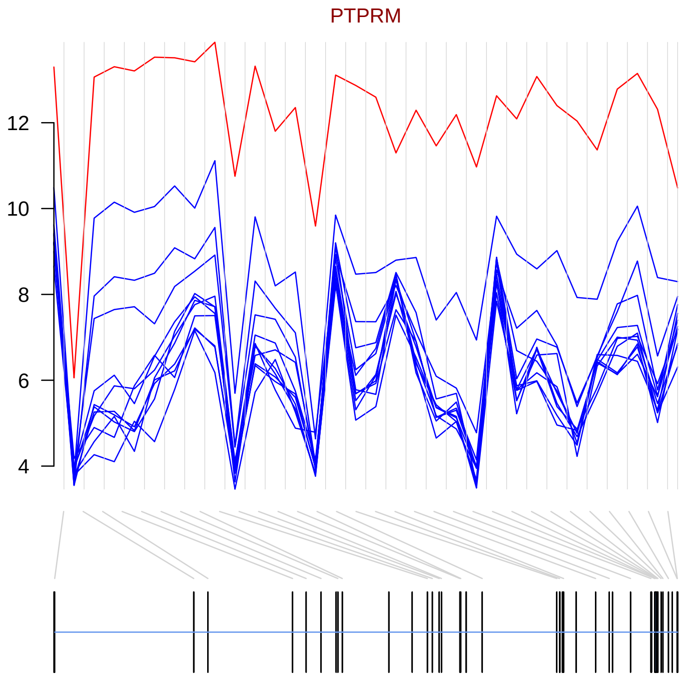

1.11 Analysis of sample T119

```
> .annotated.ranks <- function(mat, ordering)
+ {
+         temp.tab <- data.frame(mps=rownames(mat)[ordering], rank=1:length(ordering),
+                  stringsAsFactors=FALSE)
+         temp.tab.symb <- .mps.to.symbol(rownames(mat)[ordering], hsaps.ens)
+         temp.tab.col <- data.frame(do.call("rbind",
+                 lapply(split(temp.tab.symb,temp.tab.symb$mps),
+                 function(x) return(cbind(mps=unique(as.character(x$mps)),
+                         symbol=paste(unique(x$symbol),
+                 collapse=","))))),  stringsAsFactors=FALSE)
+         return(merge(temp.tab, temp.tab.col, by="mps", sort=FALSE))
+ }
> od.t119 <- .annotated.ranks(norm.exprs.meta,
+         model.res.ord.list[["od"]][1:30,"T119"])
```

```
[1] "3467351"
[1] "2364677"
[1] "3279313"
[1] "2500550"
[1] "3456805"
```

```
[1] "2316245"
[1] "2652801"
[1] "2632225"
[1] "3174967"
[1] "3798829"
[1] "3021269"
[1] "3699616"
[1] "3564620"
[1] "3891530"
[1] "3209726"
[1] "3239584"
[1] "2797393"
[1] "3401704"
[1] "3798778"
[1] "2665720"
[1] "3622386"
[1] "3282601"
[1] "2440327"
[1] "3593575"
[1] "2683763"
[1] "2427619"
[1] "2340961"
[1] "3777991"
[1] "2453307"
[1] "2339872"
```

```
> zscore.t119 <- .annotated.ranks(norm.exprs.meta,
+         model.res.ord.list[["z.score"]][1:30,"T119"])
```

```
[1] "2364677"
[1] "2652801"
[1] "3891530"
[1] "2632225"
[1] "3798829"
[1] "3798778"
[1] "3699616"
[1] "2683763"
[1] "3467351"
[1] "3279313"
[1] "3021269"
[1] "2713111"
[1] "3479355"
[1] "3732092"
[1] "3341798"
[1] "2440327"
[1] "3890333"
```

```
[1] "2500667"
[1] "3174967"
[1] "2790324"
[1] "3175274"
[1] "2716467"
[1] "2337716"
[1] "3140478"
[1] "2378325"
[1] "2500550"
[1] "2451567"
[1] "2339872"
[1] "2842707"
[1] "2522247"

> od.t119$rank[od.t119$symbol == "PBX1"]

[1] 2

> zscore.t119$rank[zscore.t119$symbol == "PBX1"]

[1] 1

> od.t119$rank[od.t119$symbol == "ANKS1B"]

[1] 1

> zscore.t119$rank[zscore.t119$symbol == "ANKS1B"]

[1] 9

> od.t119$rank[od.t119$symbol == "WNT16"]

[1] 11

> zscore.t119$rank[zscore.t119$symbol == "WNT16"]

[1] 11

> od.t119$rank[od.t119$symbol == "ROR1"]

[1] 30

> zscore.t119$rank[zscore.t119$symbol == "ROR1"]

[1] 28

>
```

1.12 Analysis of samples 09206 and 08419

Note that Additional_File_3.xlsx was saved as a tab delimited file

```
> samp.annots <- read.delim("../Additional_File_3.txt", sep="\t", header=TRUE, skip=4)
> des.mat <- model.matrix(~factor(Gender), data=samp.annots)
> stopifnot(all(samp.annots$SampleID == colnames(norm.exprs.meta)))
> basic.fit <- lm.fit(x=des.mat, y=t(norm.exprs.meta))
> norm.resid <- t(basic.fit$residuals)
> z.resid.mat <- zscore.matrix(norm.resid)
> od.resid.mat <- od.matrix(norm.resid, add.params=list(od.k=6))
> method.list <- list(z.mat=z.resid.mat, od=od.resid.mat)
> method.ord.list <- lapply(method.list, function(y) sapply(colnames(y),
+          function(x) order(y[,x], decreasing=TRUE)))
> top.od <- method.ord.list[["od"]][1:10,"09206"]
> pos.in.od <- match(top.od, method.ord.list[["od"]][,"08419"])
> cbind(`09206pos`=top.od, `09206in08419`=pos.in.od)
```

```
      09206pos 09206in08419
 [1,]    18021           29
 [2,]    20488            3
 [3,]    12570         5944
 [4,]      590         4109
 [5,]    11998         4341
 [6,]    14501         4846
 [7,]     8588         4177
 [8,]    13924          779
 [9,]     4470         7072
[10,]     4983         3109
```

```
> ranks.09206 <- .annotated.ranks(norm.exprs.meta, top.od)
```

```
[1] "3777470"
[1] "3926271"
[1] "3404530"
[1] "2345023"
[1] "3367673"
[1] "3553998"
[1] "3067478"
[1] "3491486"
[1] "2680046"
[1] "2736060"
```

```
> ranks.09206[2,]
```

```
      mps rank   symbol
2 3926271    2 TMPRSS15
```

```
> top.zscore <- method.ord.list[["z.mat"]][1:10,"09206"]
> pos.in.zscore <- match(top.zscore, method.ord.list[["z.mat"]][,"08419"])
> cbind(`09206pos`=top.zscore, `09206in08419`=pos.in.zscore)

      09206pos 09206in08419
 [1,]    18022        11425
 [2,]    14501        12648
 [3,]     8588        11895
 [4,]      590        12414
 [5,]    10812        10424
 [6,]     6455        11056
 [7,]     4983        11215
 [8,]    12571        13191
 [9,]     5021        11759
[10,]     4470        12431

> sessionInfo()

R version 3.0.1 (2013-05-16)
Platform: x86_64-apple-darwin12.4.0/x86_64 (64-bit)

locale:
[1] en_US.UTF-8/en_US.UTF-8/en_US.UTF-8/C/en_US.UTF-8/en_US.UTF-8

attached base packages:
 [1] grid      parallel  splines   stats     graphics  grDevices utils
 [8] datasets  methods   base

other attached packages:
 [1] GeneSelector_2.10.0     org.Hs.eg.db_2.9.0      pd.huex.1.0.st.v2_3.8.0
 [4] RSQLite_0.11.4          DBI_0.2-7               oligo_1.24.2
 [7] oligoClasses_1.22.0     GenomicFeatures_1.12.3  AnnotationDbi_1.22.6
[10] Biobase_2.20.1          GenomicRanges_1.12.5    IRanges_1.18.3
[13] BiocGenerics_0.6.0      GenomeGraphs_1.20.0     biomaRt_2.16.0
[16] Hmisc_3.12-2            Formula_1.1-1           survival_2.37-4
[19] plyr_1.8                reshape2_1.2.2          ggplot2_0.9.3.1

loaded via a namespace (and not attached):
 [1] affxparser_1.32.3     affyio_1.28.0         BiocInstaller_1.10.3
 [4] Biostrings_2.28.0     bit_1.1-10            bitops_1.0-6
 [7] BSgenome_1.28.0       cluster_1.14.4        codetools_0.2-8
[10] colorspace_1.2-2      dichromat_2.0-0       digest_0.6.3
[13] ff_2.2-11             foreach_1.4.1         gtable_0.1.2
[16] iterators_1.0.6       labeling_0.2          lattice_0.20-23
[19] MASS_7.3-28           munsell_0.4.2         preprocessCore_1.22.0
[22] proto_0.3-10          RColorBrewer_1.0-5    RCurl_1.95-4.1
[25] rpart_4.1-2           Rsamtools_1.12.4      rtracklayer_1.20.4
```

```
[28] samr_2.0            scales_0.2.3          stats4_3.0.1
[31] stringr_0.6.2       tools_3.0.1           XML_3.98-1.1
[34] zlibbioc_1.6.0

>
```
