# Supplementary material for: Comparison of methods to identify aberrant expression patterns in individual patients: augmenting our toolkit for precision medicine
Source: Genome Med. 2013 Nov 29;5(11):103. doi: 10.1186/gm509 (PMC3971350; doi:10.1186/gm509)
Supplement: Additional file 2 — Contains the supplemental Figures S1-S5. [file gm509-S2.docx]

**Figure S1 and S2. Effect of *k* and effect size on power and FDR for the outlying degree.**

Simulations were performed generating 10,000 genes and 20 samples from a normal distribution with mean 7 and standard deviation of 1 as described in the methods. 10,000 iterations were performed for each parameter combination. The OD method was run with the *k* parameter ranging from one to *m –* 1 in increments of 2 (X axis) and the FDR and Power was reported with respect to 10,000 iterations (Y-axis). The effect size measured in terms of unit difference is denoted by different line types. The grey areas indicate .95 confidence intervals. Note that for FDR, the estimates were very stable and the grey area is not readily observable.


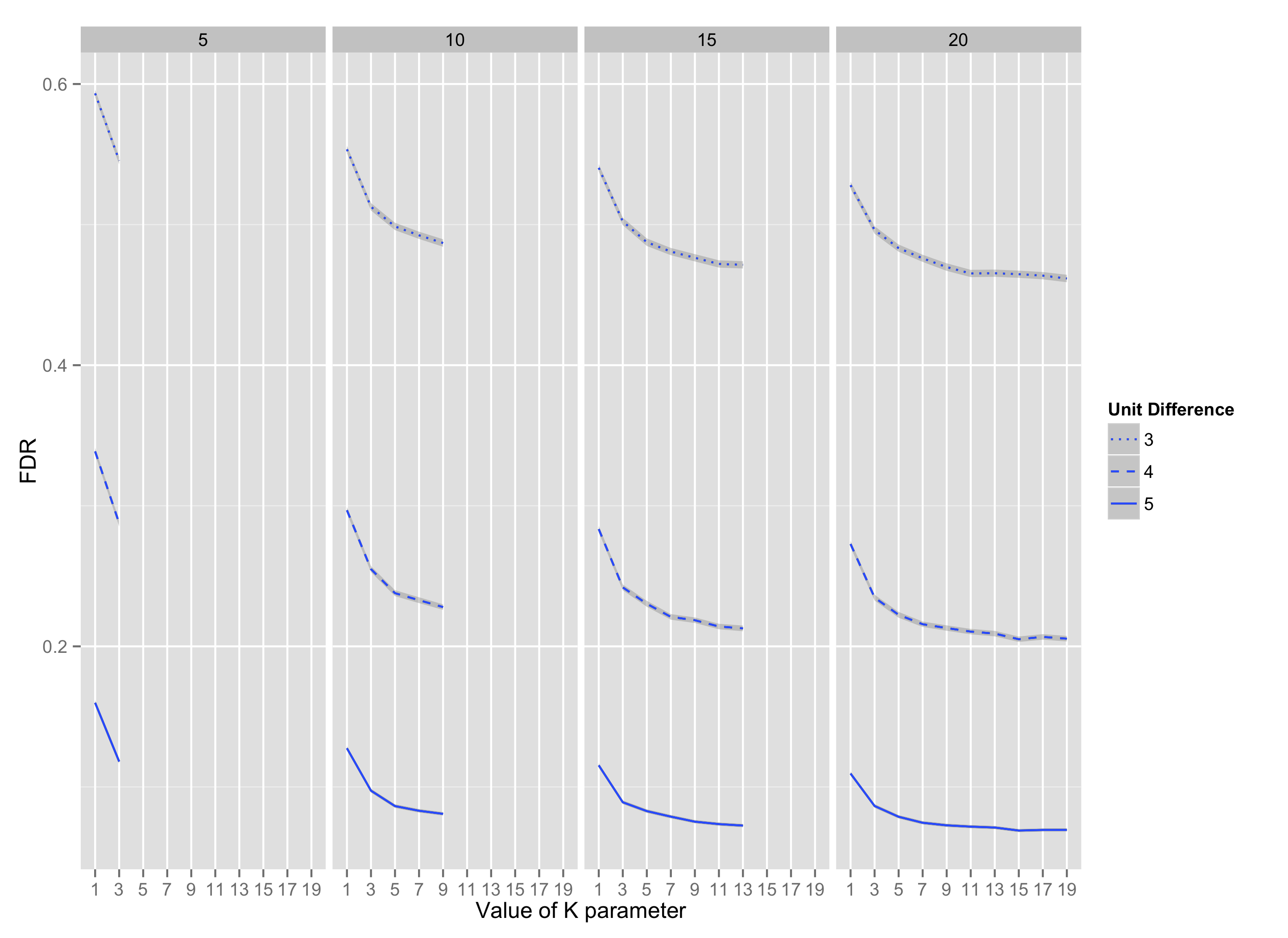

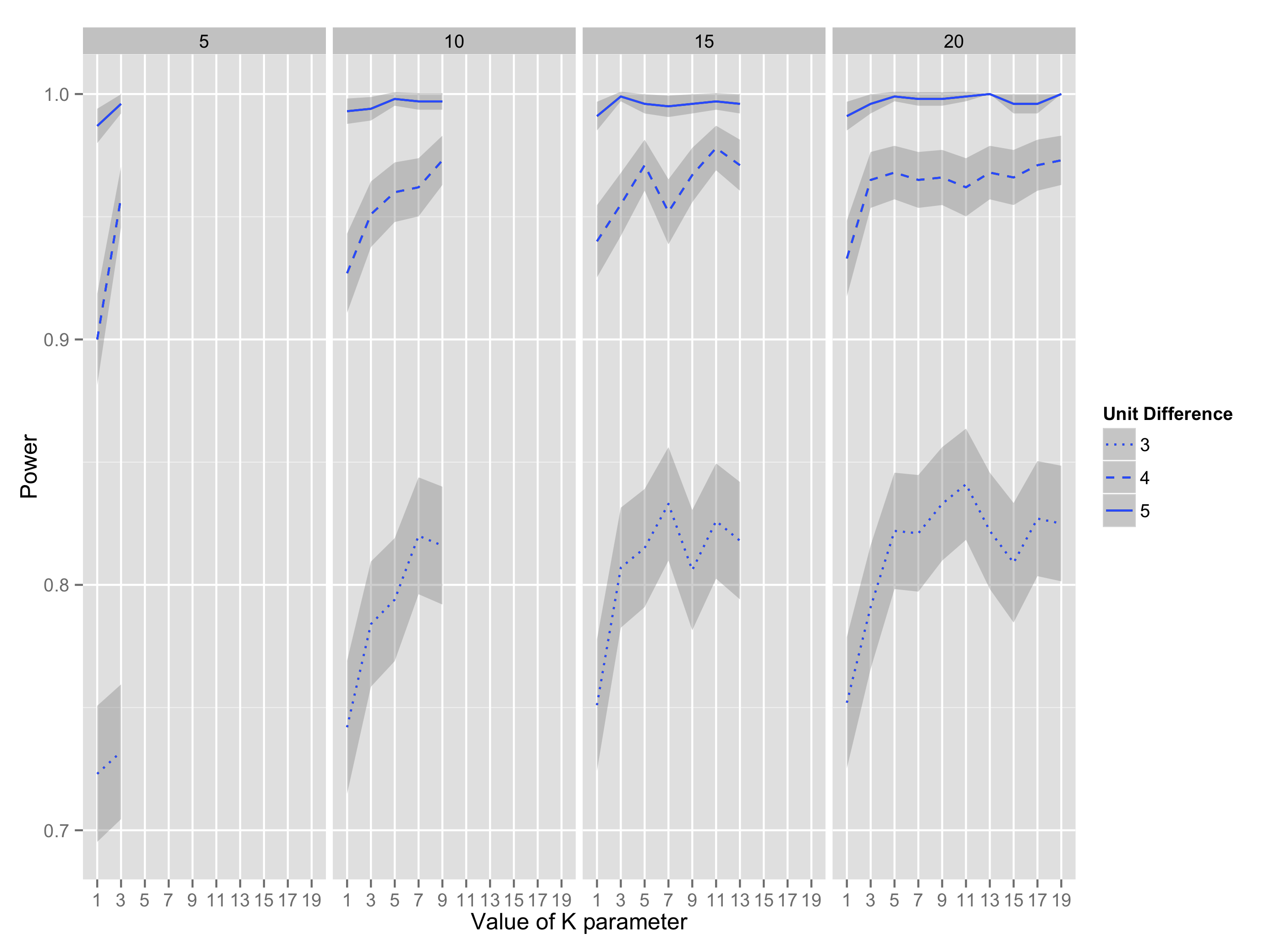


**Figure S3 and S4. Effect of *k* and effect size on power and FDR for the outlying degree relative to cohort size.**

Simulations were performed generating 10,000 genes and 20 samples from a normal distribution with mean 7 and standard deviation of 1 as described in the methods. The OD method was run with the *k* parameter ranging from one to *m –* 1 in increments of 2 (X axis) and the FDR and Power was reported with respect to 1,000 iterations (Y-axis). The effect size measured in terms of unit difference is denoted by different line types. The grey areas indicate .95 confidence intervals. Each subplot within the figures indicates a different number of samples in the assessed cohort ranging from 5-20 in intervals of 5. Note that for FDR, the estimates were very stable and the grey area is not readily observable. The Power calculations are not as stable as in Figure S1 due to 1/10 the number of iterations performed at each parameter combination.


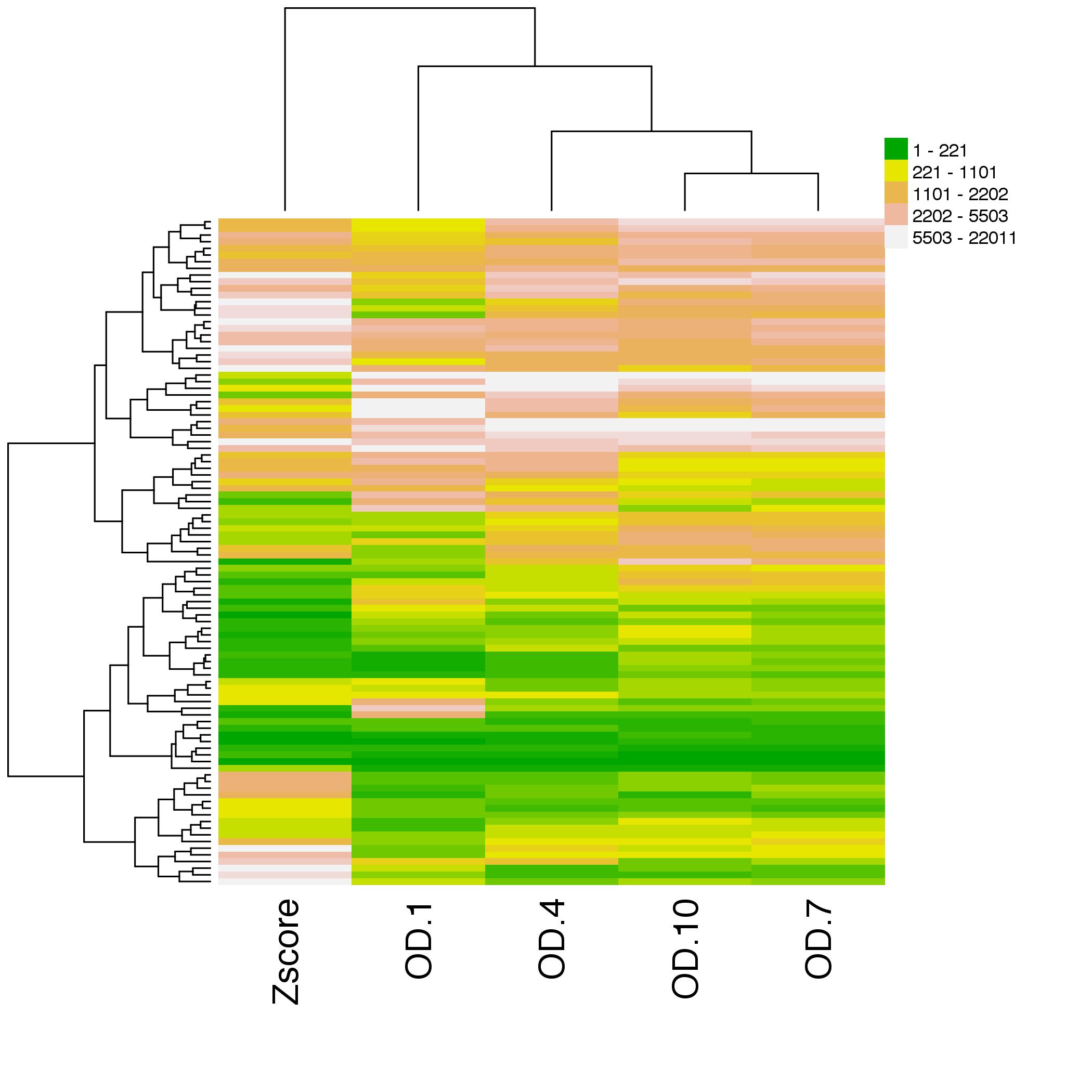


**Figure S5. Lower *k* for the OD method increases similarity of gene ranks to the Zscore method**

A heatmap is displayed based off of the comparative rankings of the OD methods with varying *k* parameters relative to the Zscore for the first 100 genes from the GeneSelector package [1]. Both the genes and methods are clustered using complete linkage hierarchical clustering and heatmap coloring is based off of the corresponding ranks. The methods are displayed at the bottom with OD displayed along with its *k* value.

**References**

1. Slawski M, Boulesteix. A: *GeneSelector: Stability and Aggregation of ranked gene lists:* ; 2009.
